# Breast Plasmacytoma as the Initial Manifestation of Multiple Myeloma in a 36-Year-Old Woman

**DOI:** 10.7759/cureus.87929

**Published:** 2025-07-14

**Authors:** Maria João Silva, Janki Patel, Alice Huang, Roberto Lo Gullo, Katja Pinker-Domenig

**Affiliations:** 1 Radiology, Unidade Local de Saúde de Trás-os-Montes e Alto Douro, Vila Real, PRT; 2 Breast Radiology, Columbia University Irving Medical Center, New York, USA

**Keywords:** breast mass, breast plasmacytoma, extramedullary plasmacytoma, mammography, multiple myeloma, ultrasonography

## Abstract

Extramedullary plasmacytomas (EMPs) of the breast are extremely rare and may present as the initial manifestation of multiple myeloma (MM). We report the case of a 36-year-old woman who presented with a rapidly growing right breast mass. Mammography and ultrasound revealed an oval, circumscribed, heterogeneous, vascular mass, measuring up to 4.2 cm, categorized as Breast Imaging Reporting and Data System (BI-RADS) 4. Core needle biopsy revealed a plasmablastic/plasmacytic neoplasm with strong CD138 expression, lambda light chain restriction, high Ki-67 index, and negative Epstein-Barr virus early RNA in situ hybridization (EBER-ISH), consistent with plasmablastic plasmacytoma. Systemic staging confirmed the diagnosis of MM, and the patient underwent chemotherapy followed by autologous stem cell transplantation. This case illustrates how breast plasmacytoma (BP) can mimic primary breast malignancies on imaging, particularly triple-negative invasive ductal carcinoma, lymphoma, or malignant phyllodes tumor. Although rare, it should be considered in younger patients with atypical breast masses. Diagnosis relies on biopsy with histopathological and immunophenotypic confirmation. Early recognition and biopsy are key, as BP may mimic aggressive breast malignancies and carry prognostic significance in MM.

## Introduction

Plasmacytomas are clonal proliferations of plasma cells that may occur in bone or soft tissues. Extramedullary plasmacytomas (EMPs), which arise outside the bone marrow, account for approximately 4% of all plasma cell neoplasms and are most frequently located in the upper aerodigestive tract [1-3]. Although extremely rare, the breast can also be affected, a presentation known as breast plasmacytoma (BP) [4].

Multiple myeloma (MM) is a malignant plasma cell disorder typically associated with anemia, hypercalcemia, renal dysfunction, and lytic bone lesions. EMPs may represent either a primary solitary lesion or, more frequently, a secondary manifestation of MM, particularly in relapsed or advanced stages [1,3,5]. MM is often diagnosed during evaluation of bone pain or fatigue [1,3]. However, in rare cases such as BP, extramedullary disease may precede these systemic symptoms, leading to diagnostic delay [4,5]. 

Clinically, BP most often presents as a solitary, unilateral palpable mass that may grow rapidly and without constitutional or systemic symptoms, particularly when it is the initial manifestation of MM [3,4]. Though less common, multiple or bilateral lesions may also occur. Given its nonspecific presentation, BP can be easily mistaken for more common breast lesions, including primary breast carcinomas or lymphomas [1,4].

BP should be considered in the differential diagnosis of solid breast masses, especially in younger patients and even in the absence of a prior diagnosis of MM, given its potential to represent the initial manifestation of systemic disease [1,3,6]. Recognizing this rare presentation is important for ensuring accurate diagnosis and appropriate management [1].

In this article, we present a case of BP as the initial manifestation of MM in a young woman, highlighting the importance of imaging-pathology correlation and the role of breast radiologists in raising suspicion for hematologic malignancies in atypical breast lesions.

## Case presentation

A 36-year-old woman with no relevant prior medical history presented with a painless right breast mass, located in the upper inner quadrant, which had grown rapidly over the previous month. On clinical examination, the lesion was firm, mobile, and measured approximately 4 cm. No associated skin changes, nipple retraction, or discharge were observed. She denied systemic symptoms such as fever, night sweats, or recent infections.

**Figure 1:**
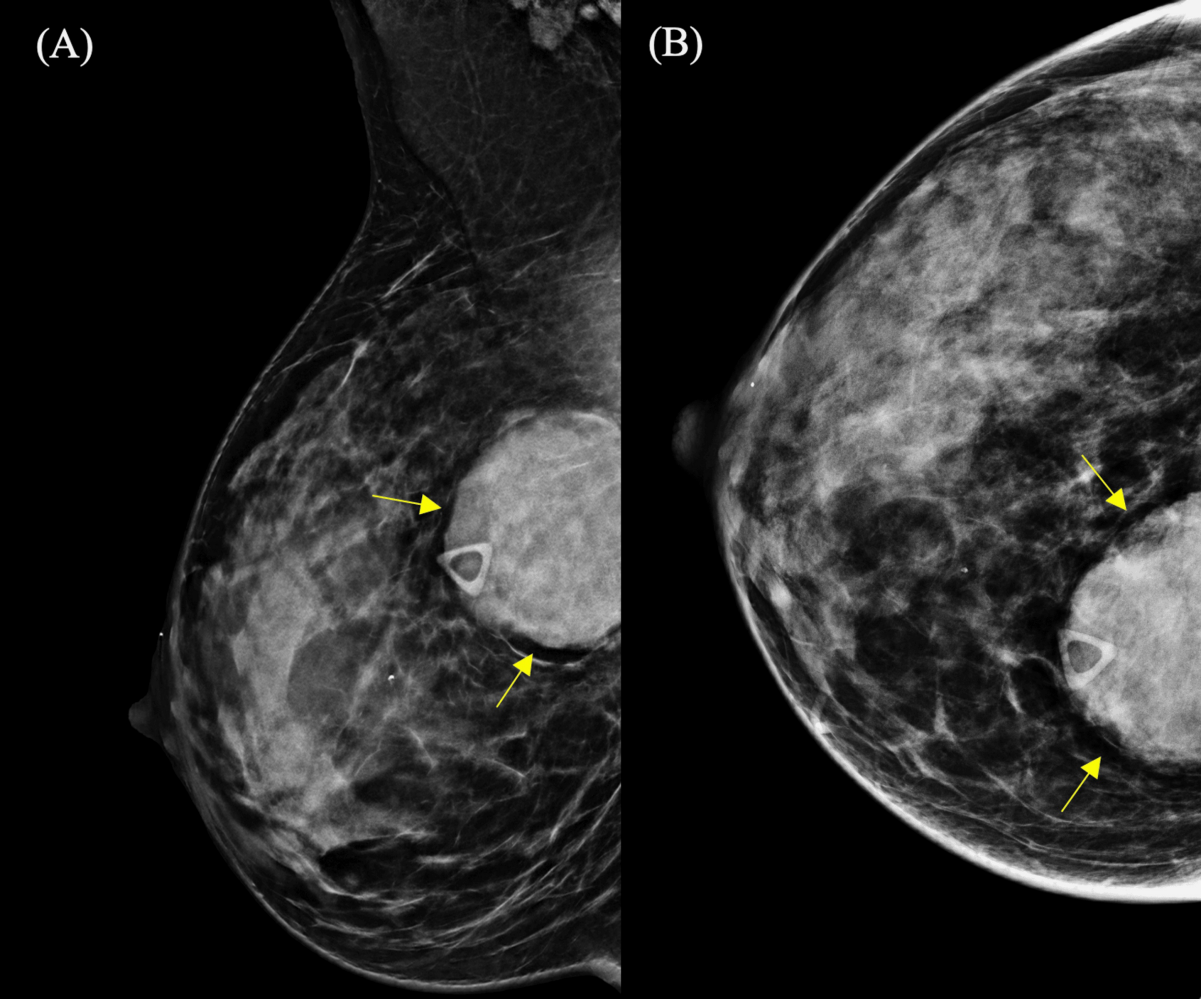
Diagnostic mammography of the right breast A. Mediolateral oblique view. B. Craniocaudal view. A triangular skin marker denotes the palpable abnormality. An oval, circumscribed, high-density mass is identified in the upper inner quadrant of the right breast at posterior depth, demonstrating a subtle partial lucent halo (arrows). No associated suspicious calcifications, architectural distortion, skin thickening, or nipple retraction are observed.

Mammography demonstrated heterogeneously dense parenchyma (Breast Imaging Reporting and Data System (BI-RADS) C) and an oval, circumscribed, high-density mass in the upper inner quadrant of the right breast, located at posterior depth. A subtle partial lucent halo was also observed. No associated microcalcifications, architectural distortion, skin thickening, or nipple retraction were identified (Figures 1A-1B).

**Figure 2:**
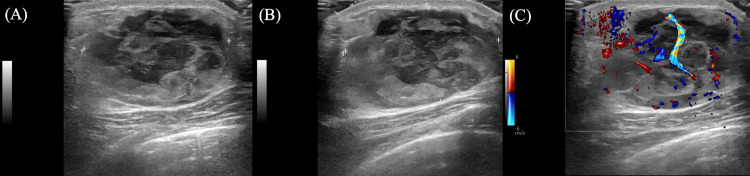
Targeted breast ultrasound of the right breast A. Grayscale ultrasound image, radial view. B. Grayscale ultrasound image, anti-radial view. C. Color Doppler image, radial view. Targeted ultrasound reveals an oval, parallel-oriented, circumscribed mass with heterogeneous echotexture, internal vascularity, and posterior acoustic enhancement, measuring 4.2 × 3.4 × 3.9 cm. The lesion is located at the 1:00 position, approximately 5 cm from the nipple.

Targeted ultrasound revealed a corresponding oval, parallel-oriented, circumscribed mass with heterogeneous echotexture, internal vascularity, and posterior acoustic enhancement, measuring 4.2 x 3.4 x 3.9 cm (Figures 2A-2C). No suspicious axillary lymphadenopathy was identified.

Given the lesion’s characteristics, it was categorized as BI-RADS 4, and an ultrasound-guided core needle biopsy was performed.

Histologic evaluation revealed a dense infiltrate of plasmablastic and plasmacytic cells with high mitotic activity. Immunohistochemistry showed strong CD138 expression, MUM1 positivity, lambda light chain restriction, and a high Ki-67 proliferation index (>80%). The neoplastic cells were negative for CD20, CD19, and Epstein-Barr virus (EBV) (Epstein-Barr virus early RNA in situ hybridization (EBER-ISH)), consistent with a diagnosis of plasmablastic plasmacytoma.

Cytogenetic analysis supported the diagnosis and indicated a high-risk myeloma subtype. In the breast lesion, fluorescence in situ hybridization (FISH) revealed a MYC rearrangement and additional MYC gene copies, along with trisomy 17. These findings suggested high proliferative activity and aggressive behavior.

Initial laboratory workup revealed mild normocytic anemia (hemoglobin: 11.2 g/dL) with normal leukocyte and platelet counts. Serum creatinine (0.62 mg/dL), calcium (8.9 mg/dL), and phosphorus (2.8 mg/dL) levels were within normal limits. Albumin was slightly decreased (3.5 g/dL), while globulin levels were elevated (7.3 g/dL), resulting in a high total serum protein (10.8 g/dL). Lactate dehydrogenase (LDH) was significantly elevated (482 U/L), and beta-2-microglobulin was mildly increased (2.9 mg/L), suggesting an underlying plasma cell dyscrasia.

**Figure 3:**
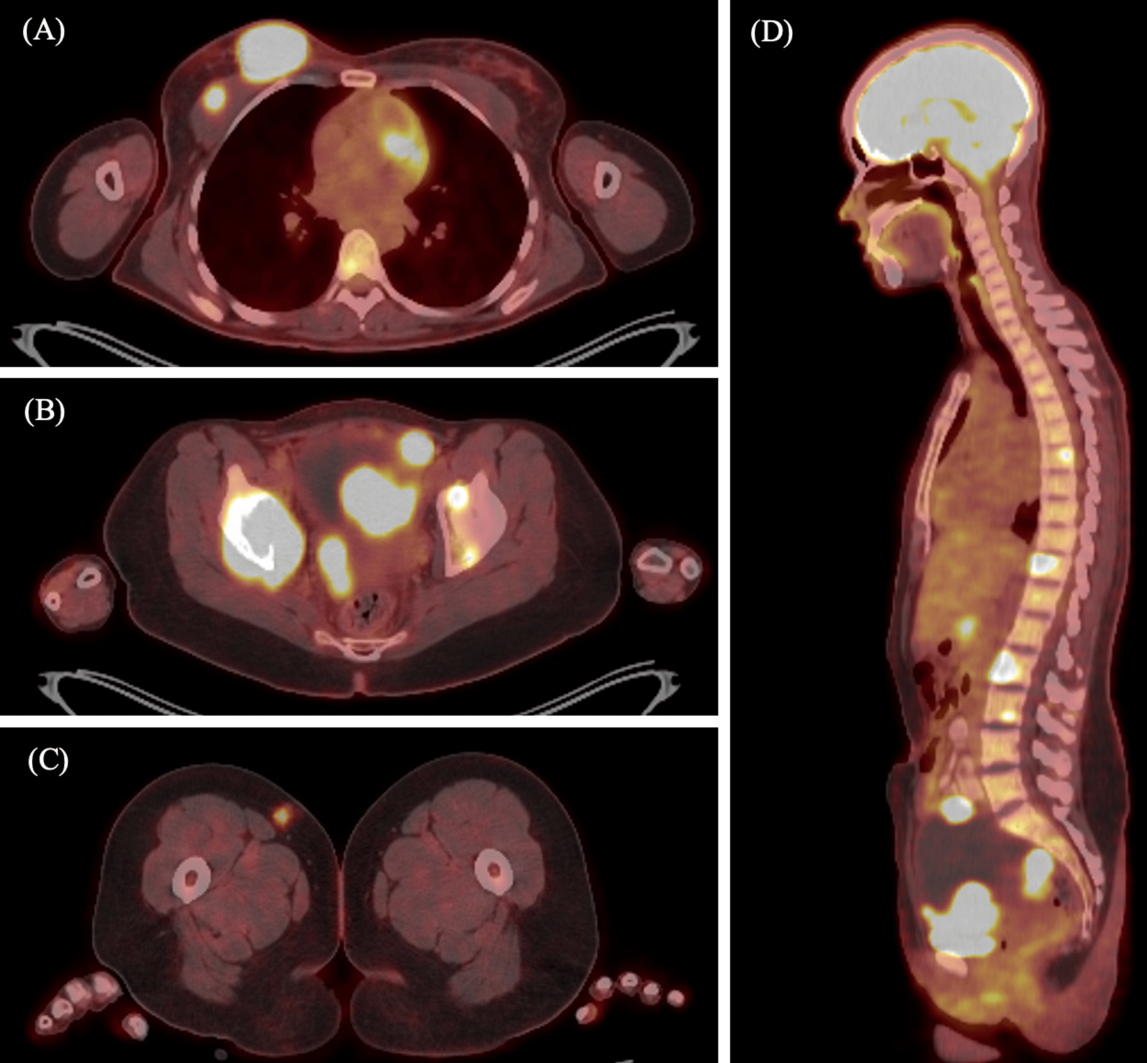
Staging FDG-PET/CT A. Thoracic axial view: FDG-avid right breast lesion and adjacent uptake in the ipsilateral pectoralis major muscle, consistent with soft tissue involvement by extramedullary plasmacytoma. B. Pelvic axial view: FDG uptake in both acetabula and femoral heads, as well as in pelvic lymph nodes and the bladder wall, indicative of widespread extramedullary disease. C. Lower limb axial view: Focal hypermetabolic subcutaneous lesion in the right thigh, suggestive of superficial soft tissue involvement. D. Sagittal view: Diffuse osseous involvement with intense FDG uptake in the thoracic and lumbar vertebrae. Additional hypermetabolic foci are noted in abdominal and pelvic soft tissues. PET/CT: positron emission tomography/computed tomography; FDG: fluorodeoxyglucose

Systemic staging with positron emission tomography/computed tomography (PET/CT) confirmed extensive disease (Figures 3A-3D). In addition to the breast lesion, multiple hypermetabolic osseous lesions were identified, along with soft tissue masses in the chest wall, bladder, and subcutaneous tissue, as well as fluorodeoxyglucose (FDG)-avid lymphadenopathy involving abdominal and pelvic chains.

Bone marrow biopsy revealed clonal plasma cell infiltration, establishing the diagnosis of MM. Cytogenetic analysis demonstrated high-risk abnormalities, including a t(4;14)(IGH::FGFR3) translocation and loss of the 3′ region of the IGH gene.

The patient was subsequently treated with systemic chemotherapy using a combination of bortezomib, lenalidomide, and dexamethasone, followed by autologous stem cell transplantation.

**Figure 4:**
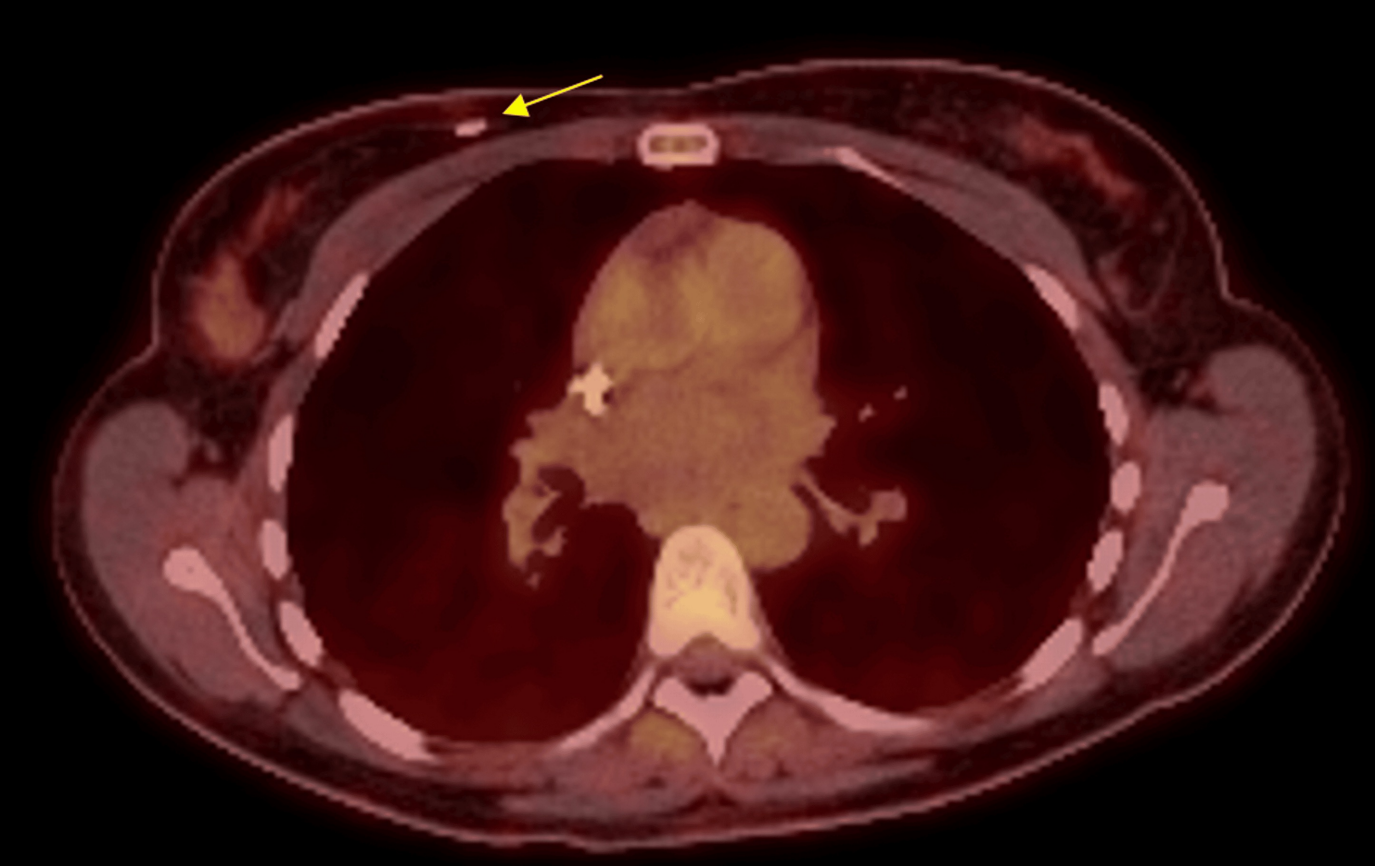
Post-treatment follow-up FDG-PET/CT Axial fused PET/CT image, thoracic level, demonstrates metabolic response of the right breast and pectoralis major lesions, with no residual FDG uptake. Only the biopsy clip remains visible (arrow), confirming a significant radiologic response following systemic therapy and autologous stem cell transplant. PET/CT: positron emission tomography/computed tomography; FDG: fluorodeoxyglucose

A follow-up PET/CT, performed three months post-treatment, demonstrated an excellent metabolic response, with complete resolution of the right breast lesion (Figure 4), soft tissue and nodal hypermetabolic lesions, as well as significant improvement in osseous disease.

## Discussion

Epidemiology and clinical significance

EMPs are rare plasma cell neoplasms that occur outside the bone marrow, accounting for approximately 4% of all cases [3]. While the upper aerodigestive tract remains the most frequent site, breast involvement is exceptionally uncommon, representing less than 2% of all EMPs and approximately 1.5% of plasmacytomas overall [4]. BP may arise as a solitary primary lesion or, more commonly, as a secondary manifestation of disseminated MM, particularly in relapsed or refractory disease [1,3,5].

Clinically, BP most often presents as a solitary, unilateral palpable mass that can grow rapidly and without systemic symptoms, leading to diagnostic challenges. This presentation is consistent with previously reported cases of BP as the first manifestation of MM [3,4] and matches the clinical scenario described in our patient.

Imaging features

Imaging features of BP are nonspecific, often mimicking more common breast malignancies [1,4]. On mammography, BP typically appears as a well-circumscribed, oval or round, high-density mass without associated microcalcifications, spiculation, or architectural distortion [1,4]. In a 13-year retrospective study, Surov et al. found that 83% of patients with plasmacytomas presented with breast lumps and 66% had a unilateral mass [4]. In this case, the lesion demonstrated these typical features and was also surrounded by a subtle partial lucent halo, a finding previously described in the literature [1].

On ultrasound, BPs have been described as oval or round lesions, with well-defined or slightly indistinct margins and heterogeneous to hypoechoic echotexture [1,4]. A peripheral hyperechoic rim may also be present and has been suggested as a useful, though nonspecific, clue [1]. These lesions frequently demonstrate internal vascularity, while posterior acoustic features may vary; in our case, no posterior shadowing was noted [1].

Magnetic resonance imaging (MRI), when performed, may demonstrate moderate to intense enhancement and internal heterogeneity, but lacks specific diagnostic criteria for BP [5]. 

Given the lesion’s rapid growth, solid and hypervascular nature, and absence of typical benign features, it was categorized as BI-RADS 4, and an ultrasound-guided core needle biopsy was performed.

Differential diagnosis

The imaging features of BP overlap with those of several benign and malignant breast lesions, requiring careful consideration of differential diagnoses. The most critical distinction is from primary breast carcinoma, particularly triple-negative subtypes, which are more common in younger patients and lack hormone receptor or HER2 expression, similar to BP. These carcinomas may present as well-circumscribed, oval, or round masses without calcifications [1]. However, subtle features such as indistinct, angular, or microlobulated margins, when present, may help differentiate them from plasmacytomas. 

Other subtypes of invasive mammary carcinoma, such as mucinous, medullary, and papillary carcinoma, as well as phyllodes tumors, especially malignant forms, are also considered in the differential diagnosis. These lesions may present as well-circumscribed, lobulated, or oval solid masses, with homogeneous or heterogeneous echotexture, and may mimic benign or low-grade tumors on ultrasound and mammography [1]. Despite their distinct histological profiles, their imaging overlap with BP supports their inclusion in the differential diagnosis.

Primary breast lymphoma, particularly diffuse large B-cell lymphoma, is another important differential. These lesions may appear as well-defined, hypoechoic, noncalcified masses, similar to BP [1,3]. However, they lack plasma cell markers such as CD138 and do not exhibit light chain restriction, which are key distinguishing features on immunohistochemistry [1].

Fibroadenomas are also considered due to their common occurrence and benign appearance: oval, circumscribed, hypoechoic masses with posterior enhancement. However, their homogeneous texture and slow growth help distinguish them from BP [1].

Plasmablastic lymphoma may closely resemble plasmablastic myeloma, including on immunohistochemistry. However, it typically expresses EBV, demonstrated by EBER-ISH positivity, and may retain B-cell markers like CD20 or CD79a, which are usually absent or only weakly expressed in plasmacytomas [7,8].

In our patient, the absence of epithelial markers, strong CD138 positivity, lambda light chain restriction, and negative EBER-ISH were essential in confirming the diagnosis of plasmablastic plasmacytoma.

Histopathological and immunophenotypic confirmation

Given the imaging overlap with other breast lesions, histologic and immunohistochemical evaluation are essential for definitive diagnosis. Plasmablastic plasmacytomas typically exhibit high mitotic activity and a diffuse infiltrate of plasmablastic or plasmacytic cells [2,3]. In our case, the lesion demonstrated strong CD138 and MUM1 positivity, lambda light chain restriction, a high Ki-67 proliferation index (>80%), and negativity for CD20, CD19, and EBER-ISH; findings consistent with a plasmablastic plasmacytoma rather than lymphoma or carcinoma [1,2,7,8]. 

Systemic staging and prognostic implications

Further systemic evaluation with PET/CT and bone marrow biopsy is mandatory to distinguish solitary BP from systemic MM. In this case, staging revealed multiple extramedullary lesions and bone marrow involvement, confirming the diagnosis of MM. Additional laboratory workup supported the plasma cell dyscrasia, with elevated LDH and beta-2 microglobulin levels, mild anemia, and a reversed albumin-to-globulin ratio. Cytogenetic analysis revealed high-risk abnormalities, including MYC rearrangement and t(4;14)(IGH::FGFR3) translocation, both of which have been associated with aggressive clinical behavior and poorer prognosis [9,10].

The presence of EMPs in the context of MM is associated with reduced progression-free and overall survival [3,5]. In some cohorts, extramedullary relapse has been linked to a median survival as low as 4-6 months [5,8]. However, early diagnosis and timely treatment, particularly in transplant-eligible patients, can significantly improve outcomes [3].

Management considerations

Solitary plasmacytomas may be effectively treated with local radiotherapy. In the context of MM, systemic chemotherapy remains the cornerstone of treatment, typically involving regimens with bortezomib, lenalidomide, and dexamethasone, followed by autologous stem cell transplantation when feasible [3]. Surgical excision is rarely indicated, and unnecessary mastectomies should be avoided, particularly in younger women, highlighting the importance of accurate diagnosis and interdisciplinary management [1,3].

In this case, the diagnosis of MM with extramedullary breast involvement was established promptly, allowing the timely initiation of systemic therapy with a bortezomib-based regimen, with good clinical response to date.

## Conclusions

BP is a rare manifestation of plasma cell neoplasms that may present as a solitary breast lesion or signal the onset of systemic MM. Given its nonspecific imaging appearance, often resembling both primary breast malignancies and hematologic conditions such as lymphoma, definitive diagnosis requires histopathological and immunophenotypic confirmation. Plasmacytoma should be considered in the differential diagnosis of rapidly enlarging solid breast masses, even in patients without a known history of MM, particularly when imaging findings are atypical. A comprehensive workup, including imaging, biopsy, and systemic staging, is essential to distinguish this entity from primary breast cancer and to guide appropriate oncologic treatment. Timely recognition is critical, as extramedullary involvement in MM portends a worse prognosis. Coordinated multidisciplinary evaluation ensures accurate diagnosis and facilitates optimal clinical management.
